# Supplementary material for: The Road towards Gene Therapy for X-Linked Juvenile Retinoschisis: A Systematic Review of Preclinical Gene Therapy in Cell-Based and Rodent Models of XLRS
Source: Int J Mol Sci. 2024 Jan 19;25(2):1267. doi: 10.3390/ijms25021267 (PMC10816913; doi:10.3390/ijms25021267)
Supplement: Supplementary file 1 [file ijms-25-01267-s001.zip › ijms-2813908-supplementary.pdf]

International Journal of *Molecular Sciences*

MDPI

Supplementary file 1: systematic review protocol.

1. * Review title (working title).

Gene therapy in rodent and cell models for X-Linked Retinoschisis: a systematic review.

2. Original language title.

n/a

3. * Anticipated or actual start date.

01 May 2021

4. * Anticipated completion date.

To be determined

5. * Stage of review at time of protocol writing.

Review stage	Started	Completed
Preliminary searches	YES	YES
Piloting of the study selection process	YES	YES
Formal screening of search results	YES	NO
Data extraction	NO	NO
Risk of bias (quality) assessment	NO	NO
Data analysis	NO	NO

6. * Named contact.

Isa van der Veen

Email salutation (e.g. "Dr Smith" or "Joanne") for correspondence: Isa

7. * Named contact email.

i.vanderveen@amsterdamumc.nl

8. Named contact address

Amsterdam UMC loc. AMC

Meibergdreef 9

1105 AZ Amsterdam

The Netherlands

9. Named contact phone number.

n/a

10. * Organisational affiliation of the review.

Amsterdam University Medical Centre, University of Amsterdam

Organisation web address: https://www.amc.nl/web/home.htm

11. * Review team members and their organisational affiliations.

Isa van der Veen, MSc. Department of Ophthalmology, Amsterdam UMC, The Netherlands

Andrea Heredero Berzal, MSc, Department of Ophthalmology, Amsterdam UMC, The Netherlands

Céline Koster, MSc, Department of Human Genetics, Amsterdam UMC, The Netherlands

Dr. ir. Anneloor L.M. ten Asbroek, Department of Human Genetics, Amsterdam UMC, The Netherlands

Prof. Dr. Arthur A.B. Bergen, Department of Human Genetics, Amsterdam UMC, The Netherlands

Prof. Dr. Camiel J.F. Boon, Department of Ophthalmology, Amsterdam UMC, The Netherlands

12. * Funding sources/sponsors.

ZonMW VIDI grant

Grant number: 09150171910048

13. * Conflicts of interest.

None

14. Collaborators.

None

15. * Review question.

Main question:

What is the current status of genetic therapy research in X-Linked Juvenile Retinoschisis disease models?

Subquestions:

Which *in vivo* and *in vitro* models exist for X-Linked Juvenile Retinoschisis?

Which therapies have been tested in these models?

Specifically, which methods of gene therapy have been tested in XLRS models and how do they impact disease parameters?

At what stage of disease is therapy most effective?

Are *in vitro* results predictive of *in vivo* results, in studies with a similar intervention design? Does this extend to clinical results?

16. * Searches.

We will search Medline through PubMed, EMBASE and Web of Science. Furthermore, we will search for grey literature and conference proceedings. We will not restrict our search on publication date or language. Prior to final analysis, we will re-run the search.

The full search strategy will be provided.

17. URL to search strategy.

To be determined.

18. * Condition or domain being studied.

X-linked Juvenile Retinoschisis (XLRS) is an ocular disease affecting young boys from childhood onwards. It is characterized by spokewheel-patterned schisis in the macula of the retina. This leads to marked visual impairments and greatly affects quality of life. It is caused by mutations in the gene encoding the retinoschisin-1 (RS1) gene. To date, there is no therapy available.

19. * Participants/population.

Inclusion criteria:

Any *in vitro* model for XLRS (iPSC-derived cell lines, organoids etc.)

Any animal model for XLRS (rat, mouse, etc.)

Clinical trials for (genetic) therapy in XLRS.

Exclusion criteria:

Not modeling the disease of interest.

20. * Intervention(s), exposure(s).

Inclusion criteria:

Any intervention to treat XLRS.

Exclusion criteria:

None

21. * Comparator(s)/control.

Inclusion criteria:

Any suitable control group.

Exclusion criteria:

No control group, unsuitable control group.

22. * Types of study to be included.

Inclusion criteria:

All types of studies are eligible.

Exclusion criteria:

None

23. Context.

Inclusion criteria:

All.

Exclusion criteria:

None.

24. * Main outcome(s).

Inclusion criteria:

Any functional or morphological outcome related to vision

Exclusion criteria:

None

25. * Additional outcome(s).

n/a

26. * Data extraction (selection and coding).

Studies will be screened for inclusion in two phases. In phase one, all studies will be screened by title and abstract. According to our predefined selection criteria, studies will be included or excluded. This will be done by two independent reviewers. In case of disputes, they will discuss and, if needed, involve a third reviewer to arbitrate. We will use Rayyan software for the study selection.

For data extraction, we will extract the following study characteristics:

- o Authors, year of publication, journal
- o Species and strain OR cell line / model
- o Group size, sex, age, weight OR culture conditions
- o Genetic modification / mutation of interest
- o Therapy type
- o Delivery method (e.g. subretinal injection)
- o Vector type (e.g. AAV-vector)
- o Timing of therapy, number of deliveries

For outcome data:

Electroretinogram (ERG) outcomes:

a-, b- and c-wave amplitudes (continuous; Volts).

Behavioural outcomes:

Performance in vision-related tasks (continuous; any reported unit of measurement)

Morphology:

Optical coherence tomography (OCT):

Thickness of retinal layers, as well as the retina overall (continuous; µm), presence of cavities (dichotomous), cavity size (continuous, µm)

Immunohistochemistry (IHC)

Number of photoreceptors (continuous; number of cells/mm2), quantification of RS1 expression.

Expression of delivered gene (mRNA, protein)

PCR (dichotomous) or qPCR (continuous; fold change)

Western blot (dichotomous or continuous)

Data will be gathered by extracting number from text and tables, as well as from graphs using a digital screen ruler. We will contact authors by email for missing or unreported data. Data will be recorded in an Excel spreadsheet. Data extraction will be done by two reviewers and randomly sampled to check for inaccuracies.

27. * Risk of bias (quality) assessment.

Using the SYRCLE method, we will perform a risk of bias assessment. Two reviewers will be involved in the assessment. In case of disagreements, a third reviewer will be involved. Risk of bias will be done at study level. Results will influence the discussion of the data synthesis.

28. * Strategy for data synthesis.

For all outcome measures listed above, a meta-analysis will be performed if there are enough (more than 2) similar papers reporting on this outcome measure. We will use standardized mean differences for continuous data and risk ratios for dichotomous. We will use a random effects model to analyze the data and I^2 to assess heterogeneity.

29. * Analysis of subgroups or subsets.

We will have subgroup analyses per intervention type per model type (mouse or organoid etc) if enough studies are available. Residual R^2

30. * Type and method of review.

Type of review

Intervention, meta-analysis, pre-clinical, systematic review

Health area of the review

Eye disorders, genetics

31. Language.

English.

32. * Country.

The Netherlands.

33. Other registration details.

N/A

34. Reference and/or URL for published protocol.

To be determined

35. Dissemination plans.

Upon completion, we intend to publish the review.

36. Keywords.

Systematic review; meta-analysis; X-Linked Juvenile Retinoschisis; disease models; macular degeneration

37. Details of any existing review of the same topic by the same authors.

None.

38. * Current review status.

Review ongoing.

39. Any additional information.

None.

40. Details of final report/publication(s).

This field should be left empty until details of the completed review are available.

Supplementary file 2: Systematic search strings

Database(s): Ovid MEDLINE(R) ALL 1946 to May 28, 2021

#	Searches	Results
**1**	exp Retinoschisis/	780
**2**	("x link* retinoschisis" or XLR* or retinoschisin or XRS or RS1 or ((pediatric or juvenile or congenital or "x link*") adj5 ("x link* retinoschisis" or retinoschisis))).ti,ab,kf.	2060
**3**	((inherit* or hereditary or genetic*) adj4 retinoschisis).ti,ab,kf.	78
**4**	or/1-3	2521
**5**	exp Genetic Therapy/ or exp Genetic Engineering/ or exp Gene Editing/ or exp CRISPR-Cas Systems/ or exp DNA Repair/ or exp Gene Transfer Techniques/ or exp Gene Transfer, Horizontal/ or exp Genetic Vectors/ or exp Transduction, Genetic/	482466
**6**	((gene* or genomic* or genetic* or DNA or nucleotide* or base* or prime* or mutation*) adj4 (rescue or repair* or therap* or edit* or replac* or correct* or engineer* or tranfer* or transduction* or supplement*)).ti,ab,kf.	378141
**7**	((dependovirus or adenovirus or adeno associated virus* or AAV) adj15 ((gene or genomic* or genetic* or DNA) adj4 (supplement* or deliver* or repair* or therap* or edit* or replac* or correct* or engineer* or tranfer* or transduction))).ti,ab,kf.	7092

**8**	((viral or virus* or AAV or adeno associated) adj5 (vector* or transduction*)).ti,ab,kf.	29893
**9**	(CRISPR or CRISPR-Cas).ti,ab,kf.	23646
**10**	exp Embryoid Bodies/ or exp Embryonic Stem Cells/ or exp Pluripotent Stem Cells/ or exp Organoids/	50071
**11**	(pluripotent stem cell* or embryonic stem cell* or stem cell* or iPSC* or iPS* or hiPSC* or embryoid bod* or organoid* or mini-organ* or organogenesis).ti,ab,kf.	390745
**12**	exp Rats/ or exp mice/ or exp models, animal/ or exp Animal Experimentation/	3253583
**13**	(mice* or mus or mouse* or murine or rat* or murinae or rodent* or rodentia).ti,ab,kf.	7195601
**14**	((mice* or mus or mouse* or murine or rat* or murinae or rodent* or rodentia) adj3 (model* or experiment* or knockout)).ti,ab,kf.	495886
**15**	(animal adj3 (model* or experiment* or disease model*)).ti,ab,kf.	227091
**16**	(("primary" or "ex vivo" or "in vivo" or "in vitro") adj3 (cell* or culture* or model* or experiment*)).ti,ab,kf.	529702
**17**	or/5-16	8912232
**18**	4 and 17	878

2

Database(s): Embase Classic+Embase 1947 to 2021 May 28

#	Searches	Results
1	exp retinoschisis/	1917
2	("x link* retinoschisis" or XLR* or retinoschisin or XRS or ((pediatric or juvenile or congenital or "x link*") adj5 ("x link* retinoschisis" or retinoschisis))).ti,ab,kw.	2157
3	((inherit* or hereditary or genetic*) adj4 retinoschisis).ti,ab,kw.	105
4	or/1-3	3335
5	exp gene therapy/ or exp genetic engineering/ or exp clustered regularly interspaced short palindromic repeat/ or exp gene transfer/ or exp viral gene delivery system/ or exp genetic transduction/ or exp gene replacement therapy/ or exp DNA repair/ or exp gene editing/ or exp CRISPR Cas system/	771639
6	((gene* or genomic* or genetic* or DNA or nucleotide* or base* or prime* or mutation*) adj4 (rescue or repair* or therap* or edit* or replac* or correct* or engineer* or tranfer* or transduction* or supplement*)).ti,ab,kw.	529232

**7**	((dependovirus or adenovirus or adeno associated virus* or AAV) adj15 ((gene or genomic* or genetic* or DNA) adj4 (supplement* or deliver* or repair* or therap* or edit* or replac* or correct* or engineer* or tranfer* or transduction))).ti,ab,kw.	11411
**8**	((viral or virus* or AAV or adeno associated) adj5 (vector* or transduction*)).ti,ab,kw.	40066
**9**	(CRISPR or CRISPR-Cas).ti,ab,kw.	32913
**10**	exp embryoid body/ or exp embryonic stem cell/ or exp pluripotent stem cell/ or exp organoid/ or exp in vitro study/ or exp primary cell culture/ or exp tissue culture/ or exp animal model/ or exp animal experiment/ or exp mouse/ or exp rat/ or exp rodent model/ or exp in vivo study/ or exp ex vivo study/	18357067
**11**	(pluripotent stem cell* or embryonic stem cell* or stem cell* or iPSC* or iPS* or hiPSC* or embryoid bod* or organoid* or mini-organ* or organogenesis).ti,ab,kw.	580582
**12**	(mice* or mus or mouse* or murine or rat* or murinae or rodent* or rodentia).ti,ab,kw.	9691349
**13**	((mice* or mus or mouse* or murine or rat* or murinae or rodent* or rodentia) adj3 (model* or experiment* or knockout)).ti,ab,kw.	714941

**14**	(animal adj3 (model* or experiment* or disease model*)).ti,ab,kw.	320430
**15**	(("in vivo" or "in vitro" or "ex vivo" or primary) adj5 (cell* or culture* or model* or experiment*)).ti,ab,kw.	911069
**16**	or/5-15	21927533
**17**	4 and 16	2623

Database: EMBASE

TITLE-ABS-KEY-AUTH ((retinoschisis OR "x link* retinoschisis" OR xlr* OR retinoschisin OR xrs) OR ((pediatric OR juvenile OR congenital OR "x link*") W/5 ("x link* retinoschisis" OR retinoschisis))) AND TITLE-ABS-KEY-AUTH (((gene* OR genomic* OR genetic* OR dna OR nucleotide*) W/4 (rescue OR repair* OR therap* OR edit* OR replac* OR correct* OR engineer* OR tranfer* OR transduction*))) OR TITLE-ABS-KEY-AUTH (((dependovirus OR adenovirus OR "adeno associated virus*" OR aav) near/15 ((gene OR genomic* OR genetic* OR dna) near/4 (repair* OR therap* OR edit* OR replac* OR correct* OR engineer* OR tranfer* OR transduction)))) OR TITLE-ABS-KEY-AUTH (crispr OR "CRISPR-Cas") OR TITLE-ABS-KEY-AUTH (("pluripotent stem cell* or embryonic stem cell*" OR "stem cell*" OR ipsc* OR ips* OR hipsc* OR "embryoid bod*" OR organoid* OR "mini-organ*" OR organogenesis)) OR TITLE-ABS-KEY-AUTH ((mice* OR mus OR mouse* OR murine OR rat* OR murinae OR rodent* OR rodentia) W/3 (model* OR experiment* OR knockout)) OR TITLE-ABS-KEY-AUTH ((("in vivo" OR "in vitro" OR "ex vivo" OR primary) W/3 (cell* OR culture* OR model* OR experiment*))) OR TITLE-ABS-KEY-AUTH (((animal) W/3 (model* OR experiment* OR "disease model*"))) AND (LIMIT-TO (DOCTYPE , "ar") OR LIMIT-TO (DOCTYPE , "cp"))	480

DONE: DedupEndNote removed 971 records, and has written 3010 records.

Supplementary table S1: experimental conditions for all experimental groups of AAV-mediated gene augmentation studies included in the analysis.

Study ID	Ref	Species	Animal model	Sex	Vector	Interven-tion age	Injection type	Injection volume	Needle size (gauge)	Dose (vector genomes/eye)	Control type	Control treatment
Bush2016a	[85]	mouse	*Rs1h*-KO	M	AAV8-scRS/IRBP-hRS	p22 (± 3)	IVI	1 μL	35	1,00E+06	internal	untreated
Bush2016b	[85]	mouse	*Rs1h*-KO	M	AAV8-scRS/IRBP-Hrs	p22 (± 3)	IVI	1 μL	35	1,00E+07	internal	untreated
Bush2016c	[85]	mouse	*Rs1h*-KO	M	AAV8-scRS/IRBP-hRS	p22 (± 3)	IVI	1 μL	35	5,00E+07	internal	untreated
Bush2016d	[85]	mouse	*Rs1h*-KO	M	AAV8-scRS/IRBP-hRS	p22 (± 3)	IVI	1 μL	35	1,00E+08	internal	untreated
Bush2016e	[85]	mouse	*Rs1h*-KO	M	AAV8-scRS/IRBP-hRS	p22 (± 3)	IVI	1 μL	35	5,00E+08	internal	untreated
Bush2016f	[85]	mouse	*Rs1h*-KO	M	AAV8-scRS/IRBP-hRS	p22 (± 3)	IVI	1 μL	35	2,50E+09	internal	untreated
Byrne2014a	[54]	mouse	*Rs1h*$^{-/Y}$	M	AAV7m8-CAG-RS1	p30	IVI	1 μL	30,5	5,00E+10	internal	untreated
Byrne2014b	[54]	mouse	*Rs1h*$^{-/Y}$	M	AAB7m8-rho-RS1	p30	IVI	1 μL	30,5	5,00E+10	internal	untreated
Byrne2014c	[54]	mouse	*Rs1h*$^{-/Y}$	M	AAVShH10-CAG-RS1	p30	IVI	1 μL	30,5	5,00E+10	internal	untreated
Byrne2014d	[54]	mouse	*Rs1h*$^{-/Y}$	M	AAV7m8-CAG-RS1	p14	IVI	1 μL	30,5	5,00E+10	internal	untreated
Byrne2014e	[54]	mouse	*Rs1h*$^{-/Y}$	M	AAV7m8-rho-RS1	p14	IVI	1 μL	30,5	5,00E+10	internal	untreated
Byrne2014f	[54]	mouse	*Rs1h*$^{-/Y}$	M	AAVShH10-CAG-RS1	p14	IVI	1 μL	30,5	5,00E+10	internal	untreated
Byrne2014g	[54]	mouse	*Rs1h*$^{-/Y}$	M	AAV7m8-CAG-RS1	p14	IVI	1 μL	30,5	5,00E+10	internal	untreated
Byrne2014h	[54]	mouse	*Rs1h*$^{-/Y}$	M	AAV7m8-rho-RS1	p14	IVI	1 μL	30,5	5,00E+10	internal	untreated
Byrne2014i	[54]	mouse	*Rs1h*$^{-/Y}$	M	AAVShH10-CAG-RS1	p14	IVI	1 μL	30,5	5,00E+10	internal	untreated
Byrne2014j	[54]	mouse	*Rs1h*$^{-/Y}$	M	AAV7m8-CAG-RS1	p14	IVI	1 μL	30,5	5,00E+10	internal	untreated
Byrne2014k	[54]	mouse	*Rs1h*$^{-/Y}$	M	AAV7m8-rho-RS1	p14	IVI	1 μL	30,5	5,00E+10	internal	untreated
Byrne2014l	[54]	mouse	*Rs1h*$^{-/Y}$	M	AAVShH10-CAG-RS1	p14	IVI	1 μL	30,5	5,00E+10	internal	untreated

Byrne2014m	[54]	mouse	*Rs1h*$^{-/Y}$	M	AAV7m8-CAG-RS1	p14	IVI	1 µL	30,5	5,00E+10	internal	untreated
Byrne2014n	[54]	mouse	*Rs1h*$^{-/Y}$	M	AAV7m8-rho-RS1	p14	IVI	1 µL	30,5	5,00E+10	internal	untreated
Byrne2014o	[54]	mouse	*Rs1h*$^{-/Y}$	M	AAVShH10-CAG-RS1	p14	IVI	1 µL	30,5	5,00E+10	internal	untreated
Byrne2014p	[54]	mouse	*Rs1h*$^{-/Y}$	M	AAV7m8-rho-RS1	p14	IVI	1 µL	30,5	5,00E+10	internal	untreated
Byrne2014q	[54]	mouse	*Rs1h*$^{-/Y}$	M	AAVShH10-CAG-RS1	p14	IVI	1 µL	30,5	5,00E+10	internal	untreated
Janssen2008a	[76]	mouse	*Rs1h*$^{-/Y}$	M/F	AAV5-mOP500-hRS1	p15	SRI	1 µL	33	4,00E+10	internal	untreated
Janssen2008b	[76]	mouse	*Rs1h*$^{-/Y}$	M/F	AAV5-mOP500-hRS1	1m	SRI	1 µL	33	4,00E+10	internal	untreated
Janssen2008c	[76]	mouse	*Rs1h*$^{-/Y}$	M/F	AAV5-mOP500-hRS1	2m	SRI	1 µL	33	4,00E+10	internal	untreated
Janssen2008d	[76]	mouse	*Rs1h*$^{-/Y}$	M/F	AAV5-mOP500-hRS1	7m	SRI	1 µL	33	4,00E+10	internal	untreated
Kjellstrom2007	[88]	mouse	*Rs1h*-KO	M	AAV(2/2)-CMV-RS1	p14	IVI	1.5 µL	33	3,50E+10	internal	untreated
Marangoni2016a	[55]	mouse	*Rs1h*-KO	M	AAV8-scRS/IRBP-hRS	3-18w	IVI	1 µL	35	2,00E+09	external	sham
Marangoni2016b	[55]	mouse	*Rs1h*-KO	M	AAV8-scRS/IRBP-hRS	3-18w	IVI	1 µL	35	2,00E+10	external	sham
Marangoni2017a	[91]	mouse	*Rs1h*-KO	M	AAV8-scRS/IRBP-hRS	p21 – 26	IVI	1 µL	35	2,50E+09	external	sham
Marangoni2017b	[91]	mouse	*Rs1h*-KO	M	AAV8-scRS/IRBP-hRS	p21 – 26	IVI	1 µL	35	2,50E+09	external	sham
Min2005a	[86]	mouse	*Rs1h*$^{-/Y}$	M	AAV5-mOP-RS1	p15	SRI	1 µL	30,5	4,00E+10	internal	untreated
Min2005b	[86]	mouse	*Rs1h*$^{-/Y}$	M	AAV5-mOP-RS1	p15	SRI	1 µL	30,5	4,00E+10	internal	untreated
Min2005c	[86]	mouse	*Rs1h*$^{-/Y}$	M	AAV5-mOP-RS1	p15	SRI	1 µL	30,5	4,00E+10	internal	untreated
Molday2006a	[87]	mouse	*Rs1h*$^{-/Y}$	M	AAV5-mOP-RS1	p15	SRI	NR	NR	4,00E+10	internal	untreated
Molday2006b	[87]	Mouse	*Rs1h*$^{-/Y}$	M	AAV5-mOP-RS1	p15	SRI	NR	NR	4,00E+10	internal	untreated
Molday2006c	[87]	mouse	*Rs1h*$^{-/Y}$	M	AAV5-mOP-RS1	p15	SRI	NR	NR	4,00E+10	internal	untreated
Ou2015	[90]	mouse	*Rs1h*-KO	M	AAV8-scRS/IRBP-hRS	p30	IVI	NR	33	2,50E+09	internal	untreated
Park2009a	[81]	mouse	*Rs1h*-KO	M	AAV8-CMV-hRSp4	7w	IVI	1.5 µL	33	7,50E+10	internal	untreated

13

Park2009b	[81]	mouse	*Rs1h*-KO	M	AAV8-CMV-hRSp4	7w	IVI	1.5 μL	33	7,50E+10	external	untreated
Park2009c	[81]	mouse	*Rs1h*-KO	M	AAV2-CMV-mRS	6-7w	IVI	1.5 μL	33	2,30E+10	external	untreated
Scruggs2022a	[83]	mouse	*Rs1h*-KO	M	AAV2/4-CMV-RS1	p60 – 90	IVI	1 μL	33	2,00E+09	internal	untreated
Scruggs2022b	[83]	mouse	*Rs1h*-KO	M	AAV2/4-CMV-RS1	p60 - 90	SRI	2 μL	30	4,00E+09	internal	untreated
Takada2008	[89]	mouse	*Rs1h*-KO	M	AAV(2/2)-CMV-RS1	p14	IVI	1.5 μL	33	2,30E+10	internal	untreated
Vijayasarathy2021	[17]	mouse	*Rs1h*-KO	M	AAV8-scRS/IRBP-hRS	p14	SRI	1 μL	35	2,00E+09	external	sham
Vijayasarathy2022a	[34]	mouse	*Rs1h*-KO	M	rAAV8-Ple155-RS1;	P14	SRI	1 μL	35	2.00E+9	internal	untreated
Vijayasarathy2022b	[34]	mouse	*Rs1h*-KO	M	rAAV2/2-Y444-miniGluR6-RS1	P14	IVI	1 μL	35	2.19E+10	unternal	untreated
Ye2015a	[82]	mouse	$Rs1h^{-/Y}$	M	rAAV2tYF-CB-RS1	NR	IVI	1 μL	NR	1,00E+09	internal	sham
Ye2015b	[82]	mouse	$Rs1h^{-/Y}$	M	rAAV2tYF-CB-RS1	NR	IVI	1 μL	NR	1,00E+09	internal	sham
Ye2015c	[82]	mouse	$Rs1h^{-/Y}$	M	rAAV2tYF-CB-RS1	NR	IVI	1 μL	NR	4,00E+09	internal	sham
Ye2015d	[82]	mouse	$Rs1h^{-/Y}$	M	AAV2tYF-CB-RS1	NR	IVI	1 μL	NR	4,00E+09	internal	sham
Ye2022	[75]	rat	*Rs1h*-KO rat (1)	M	AAV8-scRS/IRBP-hRS	p5-6	IVI	1.5 μL	35	2,00E+10	internal	untreated
Zeng2004	[70]	mouse	*Rs1h*-KO	M	AAV(2/2)-CMV-RS1	13w	SRI	2 μL	33	4,60E+10	internal	sham
Zeng2016	[84]	mouse	*Rs1h*-KO	M	AAV8-scRS/IRBP-hRS	p22 - 26	IVI	1 μL	35	2,50E+09	internal	untreated
Zeng2022	[74]	rat	*Rs1h*-KO rat (2)	M	AAV8-scRS/IRBP-hRS	p7-8	IVI	1 μL	35	2,00E+10	internal	untreated

Supplementary table 2: input parameters for all experimental groups included in the meta-analyses for a- and b-wave amplitude, and/or b/a-wave ratio.

Study ID	Animal model	Group size (n, eyes)	Control group size (n, eyes)	Vector	Intervention age	Injection type	Dose (vector genomes/eye)	Follow-up (PI)
Bush2016a	*Rs1h*-KO	25	39	AAV8-scRS/IRBP-hRS	p22 (± 3)	IVI	1.00E+06	14w (± 2)
Bush2016b	*Rs1h*-KO	26	39	AAV8-scRS/IRBP-hRS	p22 (± 3)	IVI	1.00E+07	14w (± 2)
Bush2016c	*Rs1h*-KO	21	39	AAV8-scRS/IRBP-hRS	p22 (± 3)	IVI	5.00E+07	14w (± 2)
Bush2016d	*Rs1h*-KO	24	39	AAV8-scRS/IRBP-hRS	p22 (± 3)	IVI	1.00E+08	14w (± 2)
Bush2016e	*Rs1h*-KO	24	39	AAV8-scRS/IRBP-hRS	p22 (± 3)	IVI	5.00E+08	14w (± 2)
Bush2016f	*Rs1h*-KO	26	39	AAV8-scRS/IRBP-hRS	p22 (± 3)	IVI	2.50E+09	14w (± 2)
Byrne2014a	$Rs1h^{-/y}$	5	5	AAV7m8-CAG-RS1	p30	IVI	5.00E+10	4m
Byrne2014b	$Rs1h^{-/y}$	5	5	AAV7m8-rho-RS1	p30	IVI	5.00E+10	4m
Byrne2014c	$Rs1h^{-/y}$	5	5	AAVShH10-CAG-RS1	p30	IVI	5.00E+10	4m
Byrne2014d	$Rs1h^{-/y}$	5	8	AAV7m8-CAG-RS1	p14	IVI	5.00E+10	1m
Byrne2014e	$Rs1h^{-/y}$	8	8	AAV7m8-rho-RS1	p14	IVI	5.00E+10	1m
Byrne2014f	$Rs1h^{-/y}$	5	8	AAVShH10-CAG-RS1	p14	IVI	5.00E+10	1m
Byrne2014g	$Rs1h^{-/y}$	5	8	AAV7m8-CAG-RS1	p14	IVI	5.00E+10	2m
Byrne2014h	$Rs1h^{-/y}$	8	8	AAV7m8-rho-RS1	p14	IVI	5.00E+10	2m
Byrne2014i	$Rs1h^{-/y}$	5	8	AAVShH10-CAG-RS1	p14	IVI	5.00E+10	2m
Byrne2014j	$Rs1h^{-/y}$	5	8	AAV7m8-CAG-RS1	p14	IVI	5.00E+10	3m
Byrne2014k	$Rs1h^{-/y}$	8	8	AAV7m8-rho-RS1	p14	IVI	5.00E+10	3m
Byrne2014l	$Rs1h^{-/y}$	5	8	AAVShH10-CAG-RS1	p14	IVI	5.00E+10	3m
Byrne2014m	$Rs1h^{-/y}$	5	8	AAV7m8-CAG-RS1	p14	IVI	5.00E+10	4m
Byrne2014n	$Rs1h^{-/y}$	8	8	AAV7m8-rho-RS1	p14	IVI	5.00E+10	4m
Byrne2014o	$Rs1h^{-/y}$	5	8	AAVShH10-CAG-RS1	p14	IVI	5.00E+10	4m
Byrne2014p	$Rs1h^{-/y}$	5	5	AAV7m8-rho-RS1	p14	IVI	5.00E+10	15m
Byrne2014q	$Rs1h^{-/y}$	5	5	AAVShH10-CAG-RS1	p14	IVI	5.00E+10	15m
Janssen2008a	$Rs1h^{-/y}$	5	5	AAV5-mOP-RS1	p15	SRI	4.00E+10	4m
Janssen2008b	$Rs1h^{-/y}$	5	5	AAV5-mOP-RS1	1m	SRI	4.00E+10	4m
Janssen2008c	$Rs1h^{-/y}$	5	5	AAV5-mOP-RS1	2m	SRI	4.00E+10	4m
Janssen2008d	$Rs1h^{-/y}$	5	5	AAV5-mOP-RS1	7m	SRI	4.00E+10	4m
Janssen2008d	$Rs1h^{-/y}$	5	5	AAV5-mOP-RS1	7m	SRI	4.00E+10	4m
Marangoni2017a	*Rs1h*-KO	26	29	AAV8-scRS/IRBP-hRS	p21 - 26	IVI	2.50E+09	4m
Marangoni2017b	*Rs1h*-KO	29	29	AAV8-scRS/IRBP-hRS	p21 - 26	IVI	2.50E+09	4m
Min2005a	$Rs1h^{-/y}$	15	15	AAV5-mOP-RS1	p15	SRI	4.00E+10	1m
Min2005b	$Rs1h^{-/y}$	15	15	AAV5-mOP-RS1	p15	SRI	4.00E+10	2m
Min2005c	$Rs1h^{-/y}$	15	15	AAV5-mOP-RS1	p15	SRI	4.00E+10	3m
Molday2005a	$Rs1h^{-/y}$	7	7	AAV5-mOP-RS1	p15	SRI	4.00E+09	1m
Molday2005b	$Rs1h^{-/y}$	7	7	AAV5-mOP-RS1	p15	SRI	4.00E+09	2m
Molday2005c	$Rs1h^{-/y}$	7	7	AAV5-mOP-RS1	p15	SRI	4.00E+09	3m
Park2009a	*Rs1h*-KO	8	6	AAV8-CMV-hRSp4	7w	IVI	7.50E+10	11w
Park2009b	*Rs1h*-KO	5	5	AAV8-CMV-hRSp4	7w	IVI	7.50E+10	15w
Scruggs2022a	*Rs1h*-KO	4	12	AAV2/4-CMV-RS1	p60-p90	IVI	2.00E+09	6w
Scruggs2022b	*Rs1h*-KO	4	12	AAV2/4-CMV-RS1	p60-p90	IVI	2.00E+09	6w
